# Hair Restorers

**Published:** 1869-07

**Authors:** 


### HAIR RESTORERS.

DANGER FROM THEIR USE!

Take up almost any daily paper and you will find its columns teeming with advertisements of preparations for the hair—"restorers," that will cause hair to grow upon any surface where it ever flourished before—will restore your gray hair to its natural color, and without dyeing, too—is your hair coarse and straight?—you can be supplied with an article that will render it as fine as wool, and quite as curly.

"Restorers" that will give gloss and lustre to your cherished locks, and cause them to grow even to the skirts of your garments, so that like unto the "Darby Ram," the fowls of the air can build their nests in the product of your scalp, and you be none the wiser thereof.

The sale of these hair preparations has been immense, and so extensively have they been used that we have had ample opportunities of witnessing their result both upon the hair and constitution.

Most of the hair preparations are composed of medicines which will not only injure the hair and scalp, but absolutely render the system liable to poisoning through their absorbtion.

"Mrs. S. A. Allen's World's Hair Restorer," and "Hall's Hair Restorative," seem to have the largest "run" among those who have a desire to sprout their hair, or color their locks.

Now, both these preparations owe their coloring or dyeing effects to the chemical action of acetate of lead and lac sulphur upon the hair.

Lead, when absorbed into the circulation, gives rise to neuralgic pains, great nervous irritability, and pains or cramps in stomach and bowels. We have seen at least one case of paralysis of one-half of the face, which we could trace directly to the too free use of one of the popular nostrums above mentioned.

Painters are subject to attacks of colic, with extreme prostration, from coming so constantly in contact with the "white lead" of which their paint is composed. How much more readily the poison can be absorbed when applied to the porous and sensitive scalp, our readers can readily imagine.

There are more than two dozen of these "hair restorers" offered for sale, under as many different and fanciful names, all of which have acetate of lead, lac sulphur, glycerine and water for their principal ingredients. We advise our readers to abandon their use, for no one can be healthful who is addicted to dressing their hair and scalps with such poisonous compounds.

